# Preoperative RAS Mutational Analysis Is of Great Value in Predicting Follicular Variant of Papillary Thyroid Carcinoma

**DOI:** 10.1155/2015/697068

**Published:** 2015-01-12

**Authors:** Tae Sook Hwang, Wook Youn Kim, Hye Seung Han, So Dug Lim, Wan-Seop Kim, Young Bum Yoo, Kyoung Sik Park, Seo Young Oh, Suk Kyeong Kim, Jung Hyun Yang

**Affiliations:** ^1^Department of Pathology, Konkuk University School of Medicine, 120 Neungdong-ro, Hwayang-dong, Gwangjin-gu, Seoul 143-701, Republic of Korea; ^2^Department of Surgery, Konkuk University School of Medicine, 120 Neungdong-ro, Hwayang-dong, Gwangjin-gu, Seoul 143-701, Republic of Korea; ^3^Department of Pathology, Konkuk University Medical Center, 120-1 Neungdong-ro, Hwayang-dong, Gwangjin-gu, Seoul 143-729, Republic of Korea; ^4^Department of Internal Medicine, Konkuk University School of Medicine, 120 Neungdong-ro, Hwayang-dong, Gwangjin-gu, Seoul 143-701, Republic of Korea

## Abstract

Follicular variant of papillary thyroid carcinoma (FVPTC), particularly the encapsulated subtype, often causes a diagnostic dilemma. We reconfirmed the molecular profiles in a large number of FVPTCs and investigated the efficacy of the preoperative mutational analysis in indeterminate thyroid nodules. BRAF V600E/K601E and RAS mutational analysis was performed on 187 FVPTCs. Of these, 132 (70.6%) had a point mutation in one of the BRAF V600E (*n* = 57), BRAF K601E (*n* = 11), or RAS (*n* = 64) genes. All mutations were mutually exclusive. The most common RAS mutations were at NRAS codon 61. FNA aspirates from 564 indeterminate nodules were prospectively tested for BRAF and RAS mutation and the surgical outcome was correlated with the mutational status. Fifty-seven and 47 cases were positive for BRAF and RAS mutation, respectively. Twenty-seven RAS-positive patients underwent surgery and all except one patient had FVPTC. The PPV and accuracy of RAS mutational analysis for predicting FVPTC were 96% and 84%, respectively. BRAF or RAS mutations were present in more than two-thirds of FVPTCs and these were mutually exclusive. BRAF mutational analysis followed by N, H, and KRAS codon 61 mutational analysis in indeterminate thyroid nodules would streamline the management of patients with malignancies, mostly FVPTC.

## 1. Introduction

Papillary thyroid carcinoma (PTC) is the most common follicular cell-derived tumor of the thyroid in countries with iodine-sufficient or iodine-excess diets [1]. PTC comprises more than 95% of the thyroid malignancies in Korea [2]. It appears to be biologically indolent and has an excellent prognosis (>90% survival at 20 years) [3]. More than 10 histologic variants are known, some of which are distinguished solely based on a peculiar microscopic appearance and others appear to have distinct clinical and prognostic characteristics. The follicular variant of papillary thyroid carcinoma (FVPTC) is the second most common morphologic subtype comprising 20–30% of all papillary carcinomas [3, 4]. Studies of this histologic subtype have demonstrated that certain clinicopathologic and molecular features are shared with papillary and follicular neoplasms, suggesting that the follicular variant represents a hybrid of these entities [5]. Morphologically, FVPTC may appear partially or completely encapsulated. The diagnosis of this type is based on its histologic features comprising characteristic nuclear features of PTC and exclusive or predominant follicular growth pattern without well-formed papillae. Therefore the main differential diagnoses of this tumor are follicular adenoma or follicular carcinoma. Most of the characteristic nuclear features of papillary carcinoma are present in this variant; however, other features such as nuclear crowding and pseudoinclusions are typically less abundant than classic papillary carcinoma [6]. Moreover, some cases have been reported in which nuclear features are only focally present and/or the development of classic papillary carcinoma nuclear features is suboptimal [1, 7, 8]. No consensus exists on how many nuclear features are sufficient for diagnosis and how prominent they should be and a high interobserver variability for diagnosing this variant has been reported between experienced pathologists [9, 10].

The most common and reliable diagnostic tool for evaluating thyroid nodules is fine-needle aspiration (FNA) cytology. Follicular patterned lesions including FVPTC, FTC, and FA are difficult to interpret either benign or malignant by cytology. Undoubtedly, the above characteristics of FVPTC still affect the FNA cytologic interpretation. Therefore, many FVPTCs are interpreted as “atypia of undetermined significance or follicular lesion of undetermined significance (AUS/FLUS)” [7, 11]. Indeed, more than half of the malignant cases initially diagnosed as “AUS/FLUS” were found to be FVPTC at surgical resection [7, 12].

With recent advances in the understanding of the molecular genetics of the thyroid cancer, molecular analysis can be applied as an adjunct diagnostic test to refine cytologic diagnosis. Oncogenic mutations present in FVPTC have been characterized in recent years. The BRAF mutation is a reliable marker of PTC or associated malignancy. The BRAF V600E mutation, which constitutes the vast majority of all BRAF alterations detected in the thyroid, is found in approximately 45% of PTCs in Western counties [6] and up to 90% of those in Korea [13–15]. This mutation is found in 11–31% of follicular variants [16, 17]. The BRAF K601E mutation is virtually restricted to the follicular or solid variant and is present at up to 9% [6, 18]. Several studies have reported that FVPTC showed a high frequency (26–45%) of RAS mutations [16, 19–21] and that most RAS mutations in PTCs were associated with FVPTC [6, 22–25].

We previously proposed a management guideline based on the BRAF V600E mutation status and found that performing BRAF V600E mutational analysis on FNA specimens was of great help to make a therapeutic decision of thyroid nodules when the FNA interpretation was AUS/FLU [15]. We have also reported that 61.4% of 132 FVPTCs had a point mutation in one of the BRAF V600E, BRAF K601E, or RAS oncogenes and all mutations were mutually exclusive [25]. In Korea, where most of the thyroid malignancy is papillary carcinoma and BRAF mutation is highly prevalent, considerable number of BRAF-negative indeterminate thyroid nodules would be FVPTC.

We therefore reconfirmed the molecular profiles in a large number of FVPTCs and investigated the efficacy of the preoperative mutational analysis in indeterminate thyroid nodules by evaluating the surgical results according to a management guideline based on the cytologic evaluation and mutational status.

## 2. Materials and Methods

### 2.1. Patient Population and Inclusion Criteria

Study approval was obtained from the Institutional Review Board of Konkuk University Medical Center (KUH 1210018 and KUH 1210028). Surgically resected thyroid nodules with preoperative FNA cytology were retrieved from the surgical pathology archives of the Konkuk University Medical Center from January 2009 to June 2014. All hematoxylin and eosin slides were independently reviewed and confirmed by the two endocrine pathologists to establish the histological diagnosis of FVPTC. A total of 187 cases were selected in this study. Diagnostic criteria to select FVPTC were (i) complete lack of well-formed papillae, (ii) predominant (>50%) follicular growth pattern, and (iii) the presence of characteristic nuclear PTC features. The DNAs were isolated from formalin-fixed paraffin-embedded (FFPE) tumor tissues and the mutational analysis for BRAF V600E/K601E and RAS genes was performed.

In order to confirm the efficacy of the preoperative mutational analysis in indeterminate thyroid nodules, FNA samples from thyroid nodules of AUS/FLUS category were prospectively tested for BRAF V600E/K601E and RAS mutation in the molecular diagnostic laboratory of the Department of Pathology at Konkuk University Medical Center from July 2012 to June 2014. After ultrasonography, clinically suspicious thyroid nodules were aspirated. The FNAs were categorized as benign, atypical cells of undetermined significance or follicular cells of undetermined significance (AUS/FLUS), follicular neoplasm or suspicious for follicular neoplasm (FN/SFN), suspicious for malignant cells (SMC), malignant, and nondiagnostic by the current Bethesda System for Reporting Thyroid Cytopathology [26]. Molecular analysis was performed after cytologic diagnosis was established. Informed consent for BRAF and RAS mutational analysis on FNA samples was received from all patients. BRAF mutational analysis was performed on thyroid nodules with an FNA diagnosis of AUS/FLUS and followed by RAS (NRAS codons 12, 13, and 61, HRAS codons 12, 13, and 61, and KRAS codons 12, 13, and 61) mutational analysis when BRAF mutation was negative. For the patients with FNA diagnosis of AUS/FLUS, we recommended surgery for BRAF or RAS-positive nodules or the mutation-negative solid nodules (greater than 50% solid portion) that were over 2 cm in size or any size with malignant sonographic features, including microcalcifications, an irregular margin of the nodule, parenchymal hypoechogenicity, and abnormal neck lymphadenopathy. The nodules outside above categories were removed when patients wanted to have a confirmative diagnosis. Otherwise, we regularly followed up the patients with ultrasonography. Among surgically resected cases, cases which provided matched FNA site and histologically confirmed cancer site were selected for histological confirmation. The results of the mutational analysis in AUS/FLUS cytology were correlated with the histological diagnosis.

### 2.2. Nucleic Acid Isolation

#### 2.2.1. Preparation of FFPE Tissue

Formalin-fixed paraffin-embedded (FFPE) thyroid tissue samples were manually dissected for DNA isolation. Briefly, 10 *μ*m sections of FFPE tissue samples were treated with DNA extraction buffer solution (500 mM Tris buffer, pH 8.3; 1 mM EDTA, pH 8.0; 5% Tween 20; and 100 *μ*g/mL Proteinase K), and 10% resin was added to the thyroid tissue and incubated at 56°C for a minimum of 1 hour. After the incubation, the tubes were heated to 100°C for 10 minutes, followed by centrifugation to pellet the debris, and 5 *μ*L of the supernatant was used in the polymerase chain reaction (PCR) analysis.

#### 2.2.2. Preparation of FNA Sample

**Figure 1:**
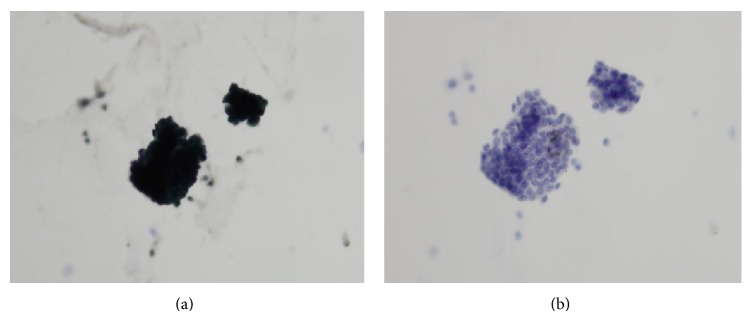
Comparison of contrast before (a) and after (b) adding extraction buffer solution.

After the coverslips were removed from the FNA slides, the atypical cells of interest were scraped and the DNA was extracted. In order to provide enough contrast to identify the target cells, we added a drop of extraction buffer on the slide (Figure 1). At least 50 cells should be dissected to have sufficient DNA. Briefly, 20–50 *μ*L of DNA extraction buffer solution (50 mM Tris buffer (pH 8.3), 1 mM EDTA (pH 8.0), 5% Tween 20, and 100 *μ*g/mL proteinase K) with 10% resin was added to the scraped cells, and this was incubated at 56°C for a minimum of 1 hour. After incubation, the tubes were heated to 100°C for 10 minutes, followed by centrifugation to pellet the debris, and 5 *μ*L of the supernatant was used in the PCR.

### 2.3. Genotype Analysis by Pyrosequencing

The PCR amplification and pyrosequencing mutational analyses were performed as described previously [14]. Each PCR mixture contained forward and reverse primers (each 0.5 pmol), 0.2 mmol of each of the dNTPs, 1.5 mmol/L MgCl_2_, 1x PCR buffer, 1.5 U of Immolase DNA polymerase (Bioline, London, UK), and 5 *μ*L of genomic DNA in a total volume of 50 *μ*L. The PCR products were electrophoresed on a 2% agarose gel to confirm successful amplification of the PCR product. The biotinylated PCR product (20 *μ*L) was attached to streptavidin-Sepharose beads (Amersham Biotechnology, Uppsala, Sweden), according to the standard protocol, by a 10-minute room temperature incubation (shaking) in binding buffer. The streptavidin-Sepharose beads were captured using a PSQ 96 sample preparation tool with 96 magnetic ejectable microcylinders (Biotage, Uppsala, Sweden). This tool was also used for the incubation (1 minute) of the biotin-streptavidin complex in 0.5 M NaOH before washing in annealing buffer. Subsequently, the samples were hybridized to 15 pmol of sequencing primers in annealing buffer at 80°C for 3 minutes followed by cooling to room temperature. The PCR primer and sequencing primer sequences are as shown in Table 1. Pyrosequencing was performed using a single-nucleotide polymorphism reagent kit (Biotage). Samples containing > 10% mutation-positive cells were considered to be positive for BRAF and RAS gene mutations.

### 2.4. Statistical Analysis

Detection of BRAF or RAS mutation in FNA samples that were histologically confirmed as malignancy or FVPTC was considered true-positive (TP), while detection of BRAF or RAS mutation in FNA samples that were histologically confirmed as benign lesion or non-FVPTC was categorized as false-positive (FP). Failure to detect BRAF or RAS mutation in the benign lesion was considered as true-negative (TN), while failure to detect BRAF or RAS mutation in the malignant lesion or FVPTC was categorized as false-negative (FN). The statistical values were calculated using the following equations:

(1) $$\begin{array}{c} \text{Sensitivity}=\left(\frac{\text{TP}}{\left(\text{TP}+\text{FN}\right)}\right)\times 100,\\ \text{Specificity}=\left(\frac{\text{TN}}{\left(\text{TN}+\text{FP}\right)}\right)\times 100,\\ \text{Positive}\;\text{predictive}\;\text{value}\;\left(\text{PPV}\right)=\left(\frac{\text{TP}}{\left(\text{TP}+\text{FP}\right)}\right)\times 100,\\ \text{Negative}\;\text{predictive}\;\text{value}\;\left(\text{NPV}\right)=\left(\frac{\text{TN}}{\left(\text{TN}+\text{FN}\right)}\right)\times 100,\\ \text{Accuracy}=\left(\frac{\left(\text{TP}+\text{TN}\right)}{\left(\text{TP}+\text{TN}+\text{FP}+\text{FN}\right)}\right)\times 100. \end{array}$$

Chi-square test was used to determine the correlation between molecular genotype and the histologic subtype of FVPTC. A *P* value of < 0.05 was considered statistically significant. SPSS 17.0 statistical software (Superior Performance Software System, SPSS for Windows; Microsoft, Chicago, IL, USA) was used for the statistical evaluation.

## 3. Results

### 3.1. Clinical and Pathological Characteristics

The clinical and pathological characteristics of the patients were listed in Table 2.

### 3.2. Mutational Status of 187 FVPTCs

Of 187 cases, 132 (70.6%) had a point mutation in one of the BRAF V600E (*n* = 57, 30.5%), BRAF K601E (*n* = 11, 5.9%), or RAS (*n* = 64, 34.2%) genes (Table 3). All mutations were mutually exclusive. The most common RAS mutations were NRAS codon 61 (*n* = 39) followed by HRAS codon 61 (*n* = 14), KRAS codon 61 (*n* = 10), and NRAS codon 12 (*n* = 1). There was no statistically significant difference in the prevalence of genotype between encapsulated and infiltrative subtypes (Table 4). Molecular genotypes according to the cytologic diagnosis were summarized in Table 5. FNA cytology was diagnosed as “nondiagnostic” (*n* = 1, 0.5%), “benign” (*n* = 8, 4.3%), “AUS/FLUS” (*n* = 89, 47.6%), “FN/SFN” (*n* = 11, 5.9%), “SMC” (*n* = 33, 17.6%), and “malignant” (*n* = 45, 24.1%). BRAF or RAS mutations are present in a significant proportion (78.7%) of FVPTC with a cytologic diagnosis in the “AUS/FLUS” category.

Of the 100 cases with an FNA diagnosis in the “AUS/FLUS or FN/SFN” category, 76 (76.0%) were positive for mutations, 19 (19.0%) for BRAF V600E, 10 (10.0%) for BRAF K601E, and 47 (47.0%) for RAS mutation. Of the 78 cases in the cytologically “SMC or malignant” category, 55 (70.5%) were positive for mutation, 38 (48.7%) for BRAF V600E, one (1.3 %) for BRAF K601E, and nine 16 (20.5%) for RAS mutation.

### 3.3. Mutational Status and Surgical Outcome of the Indeterminate Thyroid Nodules

**Figure 2:**
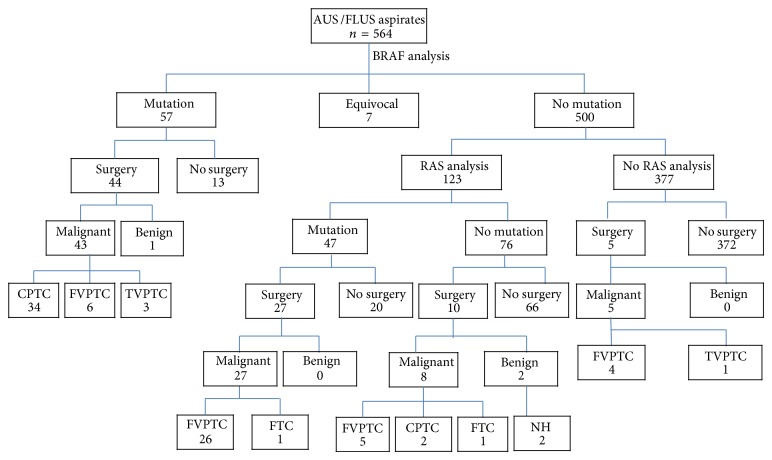
Surgical results of the 564 thyroid nodules, according to the cytologic diagnosis and BRAF V600E/K601E and RAS mutational status. CPTC: classic papillary thyroid carcinoma; FVPTC: follicular variant of papillary thyroid carcinoma; TVPTC: tall cell variant papillary thyroid carcinoma; FTC: follicular thyroid carcinoma; NH: nodular hyperplasia.

A total of 564 cases were subjected to this study (Figure 2). The correlation between BRAF or RAS mutations and surgical outcome is summarized in Figure 2. Of the 564 aspirates of AUS/FLUS category, 57 (10.1%) were positive for BRAF mutation (51 BRAF V600E and six BRAF K601E). Forty-four BRAF-positive patients underwent surgery and all except one patient were found to have PTCs (34 classic type, six follicular variant, and three tall cell variant). Of the 500 BRAF-negative aspirates, 123 underwent RAS mutational analysis. Of these 123 samples, 47 (22.0%) RAS mutations were identified, 36 NRAS codon 61, 7 HRAS codon 61, 3 KRAS codon 61, and one NRAS codon 12 (Table 6). Twenty-seven RAS-positive patients underwent surgery and all patients except one were found to have FVPTC. Of the ten RAS-negative nodules, eight were found to be malignant and two were found to be benign at surgery. The sensitivity, PPV, and accuracy of the BRAF mutational analysis for predicting malignancy were 100%, 98%, and 98%, respectively. The sensitivity, PPV, and accuracy of the BRAF mutational analysis for predicting FVPTC were 100%, 14%, and 14%, respectively. The sensitivity, specificity, PPV, NPV, and accuracy of the RAS mutational analysis for predicting malignancy were 77%, 100%, 100%, 20%, and 78%, respectively. The sensitivity, specificity, PPV, NPV, and accuracy of the RAS mutational analysis for predicting FVPTC were 84%, 83%, 96%, 50%, and 84%, respectively.

## 4. Discussion

The results of this study reconfirmed the previous findings [7, 11, 21, 24] indicating that the cytological diagnosis of FVPTC is often problematic despite the consideration of the architectural, nuclear, and background features. Thus, the diagnosis tends to be “AUS/FLUS.” Of the 187 FVPTCs with preoperative cytologic evaluation, 89 (47.6%) cases were diagnosed as “AUS/FLUS.” Therefore, a comprehensive but efficient molecular profile analysis of this tumor is required to refine the preoperative cytologic diagnosis. Among PTCs, RAS mutations are most exclusively found in follicular variants at a frequency of 26–45% [3, 4, 7, 24]. Our data supports the previous results showing that RAS mutation is present in 33.8% of the FVPTCs. The molecular profile of FVPTCs differs according to the cytologic diagnosis and this may be attributed to the morphologic heterogeneity of FVPTC. Data from our study suggest that BRAF or RAS mutations are present in a significant proportion (78.7%) of FVPTC with a cytologic diagnosis in the “AUS/FLUS” category. FVPTCs presenting low degree of cytologic features of classic PTC (AUS/FLUS or FN/SFN categories) were accompanied with a high rate of RAS mutations and a low rate of BRAF mutations. In contrast, FVPTCs with readily recognizable cytologic features of PTC (SMC or malignant categories) were harboring a high rate of BRAF mutations and a low rate of RAS mutations. These data are similar to the findings of Lee et al. [21]. There is a controversy about BRAF and RAS mutational patterns of FVPTC according to its histologic subtypes [16, 27]. This discrepancy might be attributed to the difference in defining the capsulation. The result of the present study did not show statistically significant difference in the prevalence of mutation type between encapsulated and infiltrative subtypes.

In order to confirm a preoperative adjunctive diagnostic utility of RAS mutational analysis in nodules of AUS/FLUS category, the histological diagnosis of the resected specimen was correlated with mutational status. A considerable number of BRAF-negative nodules of AUS/FLUS category were found to harbor RAS 61 mutations. Histological confirmation of 27 RAS mutation positive AUS/FLUS aspirates demonstrated that RAS mutational analysis had a high PPV of 100% for predicting malignancy, (mostly FVPTC) although the number of the cases was not sufficient. We also observed that PPV of RAS mutation for predicting FVPTC is 96% whereas that of BRAF mutation is 14%. Therefore, in clinical practice, RAS mutational analysis using FNA samples may be more useful as an ancillary test than the BRAF analysis in the diagnosis of FVPTC.

RAS mutations are not specific for malignancies, as they are present in a significant proportion of benign and malignant follicular neoplasms. Because RAS mutation is likely to predispose progression from follicular adenoma to carcinoma and to further tumor dedifferentiation, it may be justifiable to surgically remove RAS-positive adenomas to prevent progression. Some studies have even reported RAS mutation in hyperplastic nodules, by virtue of carrying a clonal mutation, but these lesions should be designated as follicular adenomas [6, 24].

Out of the three RAS genes found in thyroid cancers, the most frequently affected hot spots are NRAS codon 61 and HRAS codon 61. NRAS codon 61 mutation was the most common in the present study, followed by HRAS codon 61 and KRAS codon 61. The prevalence of KRAS mutation is higher than that in recent molecular studies [21, 24]. Interestingly, KRAS mutations detected in the present study mostly involved codon 61 but not codons 12 and 13. This finding is similar to our previous result and the result of other Korean study [21, 25]. As most of the other molecular studies in thyroid nodules included only KRAS codon 12/13 but not codon 61 [24, 28, 29], it is difficult to compare our result to the others. Whether this is attributed to the ethnic difference or not should be confirmed by larger cohort studies. We therefore suggest that KRAS mutational analysis for thyroid cancer should also include codon 61. Our data also suggest that testing for NRAS, HRAS, and KRAS codon 61 mutations can be considered sufficient for the detection of the majority of RAS mutations in FVPTCs.

## 5. Conclusions

Here we propose a cost-effective and efficient algorithm to predict FVPTC in thyroid nodules with AUS/FLUS FNA category. BRAF mutational analysis followed by N, H, and KRAS codon 61 mutational analysis in these nodules would streamline the management of patients with malignancies, mostly FVPTCs. Considering that BRAF mutation is present in more than 80% of the PTCs in Korea, RAS 61 mutation in the majority of the BRAF-negative FVPTCs, BRAF, and RAS mutations are mutually exclusive, and most FVPTCs are slow growing; an sequential algorithmic approach is more reasonable and cost-effective. The limitation of this study is that not all patients had surgical resection to confirm the molecular genotypes and the amount of RAS data with surgical outcome is too small. The replication of our findings by other groups will help to further refine the proposed management of patients with indeterminate thyroid nodules based on cytological evaluation and molecular analysis.

## Figures and Tables

**Table 1:** PCR primers for BRAF and RAS mutational analyses.

Gene	Codon		PCR primer sequences
BRAF	600/601	F	5′-B-CTTCATAATGCTTGCTCTGATAGG-3′
		R	5′-GGCCAAAAATTTAATCAGTGGAA-3′
	Sequencing		5′-CCACTCCATCGAGATT-3′

NRAS	12/13	F	5′-AGGTTCTTGCTGGTGTGAAATGAC-3′
		R	5′-B-TGGATTGTCAGTGCGCTTTTC-3′
	Sequencing		5′-TGGTGGTGGTTGGAG-3′

NRAS	61	F	5′-GATTCTTACAGAAAACAAGTGGTTATAGAT-3′
		R	5′-B-GCAAATACACAGAGGAAGCCTTCG-3′
	Sequencing		5′-GACATACTGGATACAGCTGG-3′

HRAS	12/13	F	5′-B-AGGAGCGATGACGGAATATAAGC-3′
		R	5′-TCTATAGTGGGGTCGTATTCGTCC-3′
	Sequencing		5′-TCTTGCCCACACCGC-3′

HRAS	61	F	5′-B-CCTGTTGGACATCCTGGATACC-3′
		R	5′-GTGCGCATGTACTGGTCCC-3′
	Sequencing		5′-CATGGCGCTGTACTC-3′

KRAS	12/13	F	5′-CTGGTGGAGTATTTGATAGTGTA-3′
		R	5′-B-TGGTCCTGCACCAGTAATAT-3′
	Sequencing		5′-ATAAACTTGTGGTAGTTGG-3′

KRAS	61	F	5′-B-TCCAGACTGTGTTTCTCCCTTCTC-3′
		R	5′-TACTGGTCCCTCATTGCACTGTAC-3′
	Sequencing		5′-CCTCATTGCACTGTACTC-3′

**Table 2:** Clinical and pathological characteristics of the 187 follicular variants of papillary thyroid carcinoma.

Characteristics	Number of patients (*n* = 187)	
Age, years	≤45	108
	>45	79

Gender	Female	160
	Male	27

Multiplicity	Single	142
	Multiple	45

Tumor size (cm)	≤1	120
	1 < *T* ≤ 2	41
	2 < *T* ≤ 4	21
	>4	5

Capsulation	Absent	77
	Present	110

Extrathyroidal extension	Absent	162
	Present	25

Resection margin	Clear	182
	Involvement	5

Lymph node metastases	Absent	104
	Present	25
	Not evaluated	58

Distant metastases at diagnosis	Absent	185
	Present	2

**Table 3:** The frequency of BRAF and RAS gene mutations in 187 follicular variants of papillary carcinoma.

Nucleotide change	Amino acid change	Number of mutated cases (%)
BRAF codon 600		
c.1799T>A	p.V600E	56 (29.9)
c.1799_1801delTGA	p.V600_K601del3^*^	1 (0.5)
BRAF codon 601		
c.1801A>G	p.K601E	11 (5.9)
NRAS codon 12		
c.34G>A	p.G12S	1 (0.5)
NRAS codon 61		
c.182A>G	p.Q61R	34 (18.2)
c.181C>A	p.Q61K	5 (2.7)
HRAS codon 61		
c.182A>G	p.Q61R	7 (3.7)
c.181C>A	p.Q61K	6 (3.2)
c.183G>T	p.Q61H	1 (0.5)
KRAS codon 61		
c.182A>G	p.Q61R	8 (4.3)
c.181C>A	p.Q61H	1 (0.5)
c.180_181TC>AA	p.Q61K	1 (0.5)
No mutation		55 (29.4)
Total		**187**

^*^This rare deletion mutation in the BRAF gene (c.1799_1801delTGA) converts codon 600GTG (valine) and 601AAA (lysine) to a new single codon GAA (glutamic acid).

**Table 4:** Molecular genotype according to the histologic subtypes of follicular variant of papillary thyroid carcinoma.

Genotype	Encapsulated	Infiltrative	Total	*P* value
BRAF RAS	34 (50.0%) 39 (60.9%)	34 (50.0%) 25 (39.1%)	68 64	0.207

**Table 5:** Molecular genotype according to the cytologic diagnosis of 187 follicular variants of papillary thyroid carcinoma.

	Number of BRAF V600E mutated cases (%)	Number of BRAF K601E mutated cases (%)	Number of RAS mutated cases (%)	Number of nonmutated cases (%)
Nondiagnostic (*n* = 1)	0	0	0	1 (100)
Benign (*n* = 8)	0 (0.0)	0 (0.0)	1 (12.5)	7 (87.5)
AUS/FLUS (*n* = 89)	19 (21.3)	10 (11.2)	41 (46.1)	19 (21.3)
FN/SFN (*n* = 11)	0	0	6 (54.5)	5 (45.5)
SMC (*n* = 33)	14 (42.4)	1 (3.0)	7 (21.2)	11 (33.3)
Malignant (*N* = 45)	24 (53.3)	0	9 (20.0)	12 (26.7)

Total (*N* = 187)	57 (30.5)	11 (5.9)	64 (34.2)	55 (29.4)

AUS/FLUS indicates atypia of undetermined significance or follicular lesion of undetermined significance; FN/SFN: follicular neoplasm or suspicious for follicular neoplasm; SMC: suspicious for malignant cell.

**Table 6:** The subtypes of BRAF and RAS gene mutations in 104 FNA samples of atypia of undetermined significance or follicular lesion of undetermined significance.

Nucleotide change	Amino acid change	Number of mutated cases
BRAF codon 600		
c.1799T>A	p.V600E	51
BRAF codon 601		
c.1801A>G	p.K601E	6
NRAS codon 12		
c.34G>A	p.G12S	1
NRAS codon 61		
c.182A>G	p.Q61R	29
c.181C>A	p.Q61K	7
HRAS codon 61		
c.182A>G	p.Q61R	4
c.181C>A	p.Q61K	3
KRAS codon 61		
c.182A>G	p.Q61R	2
c.180_181TC>AA	p.Q61K	1
